# Targeting Spike Glycoprotein S1 Mediated by NLRP3 Inflammasome Machinery and the Cytokine Releases in A549 Lung Epithelial Cells by Nanocurcumin

**DOI:** 10.3390/ph16060862

**Published:** 2023-06-09

**Authors:** Chuda Chittasupho, Kamonwan Srisawad, Punnida Arjsri, Rungsinee Phongpradist, Wipawan Tingya, Chadarat Ampasavate, Pornngarm Dejkriengkraikul

**Affiliations:** 1 Department of Pharmaceutical Sciences, Faculty of Pharmacy, Chiang Mai University, Chiang Mai 50200, Thailand; chuda.c@cmu.ac.th (C.C.); rungsinee.p@cmu.ac.th (R.P.); wipawan_tingya@cmu.ac.th (W.T.); chadarat.a@cmu.ac.th (C.A.); 2 Department of Biochemistry, Faculty of Medicine, Chiang Mai University, Chiang Mai 50200, Thailand; k.srisawad@gmail.com (K.S.); punnida_dream@hotmail.com (P.A.); 3 Anticarcinogenesis and Apoptosis Research Cluster, Faculty of Medicine, Chiang Mai University, Chiang Mai 50200, Thailand; 4 Center for Research and Development of Natural Products for Health, Chiang Mai University, Chiang Mai 50200, Thailand

**Keywords:** SARS-CoV-2, inflammation, nanocurcumin, A549 lung epithelial cells

## Abstract

Chronic inflammation and tissue damage can result from uncontrolled inflammation during SARS-CoV-2 or COVID-19 infections, leading to post-acute COVID conditions or long COVID. Curcumin, found in turmeric, has potent anti-inflammatory properties but limited effectiveness. This study developed nanocurcumin, a curcumin nanoparticle, to enhance its physical and chemical stability and investigate its in vitro anti-inflammatory properties upon CoV2-SP induction in lung epithelial cells. Nanocurcumin was prepared by encapsulating curcumin extract in phospholipids. The particle size, polydispersity index, and zeta potential of nanocurcumin were measured using dynamic light scattering. The encapsulated curcumin content was determined using HPLC analysis. The encapsulation efficiency of curcumin was 90.74 ± 5.35% as determined by HPLC. Regarding the in vitro release of curcumin, nanocurcumin displayed a higher release content than non-nanoparticle curcumin. Nanocurcumin was further investigated for its anti-inflammatory properties using A549 lung epithelial cell line. As determined by ELISA, nanocurcumin showed inhibitory effects on inflammatory cytokine releases in CoV2-SP-stimulated conditions, as evidenced by a significant decrease in IL-6, IL-1β and IL-18 cytokine secretions compared with the spike-stimulated control group (*p* < 0.05). Additionally, as determined by RT-PCR, nanocurcumin significantly inhibited the CoV2-SP-stimulated expression of inflammatory genes (*IL-6*, *IL-1β*, *IL-18*, and *NLRP3*) compared with the spike-stimulated control group (*p* < 0.05). Regarding the inhibition of NLRP3 inflammasome machinery proteins by Western blot, nanocurcumin decreased the expressions of inflammasome machinery proteins including NLRP3, ASC, pro-caspase-1, and the active form of caspase-1 in CoV2-SP-stimulated A549 cells compared with the spike-stimulated control group (*p* < 0.05). Overall, the nanoparticle formulation of curcumin improved its solubility and bioavailability, demonstrating anti-inflammatory effects in a CoV2-SP-induced scenario by inhibiting inflammatory mediators and the NLRP3 inflammasome machinery. Nanocurcumin shows promise as an anti-inflammatory product for preventing COVID-19-related airway inflammation.

## 1. Introduction

Severe acute respiratory syndrome coronavirus 2 (SARS-CoV-2), the primary cause of coronavirus disease 2019 (COVID-19), has resulted in a high mortality rate worldwide. Although most patients with COVID-19 have recovered within a few weeks, many continue to experience ongoing health problems, such as post-COVID-19 syndrome, long COVID-19, or post-acute sequelae of SARS-CoV-2 (PASC) [1]. The most commonly reported symptoms of post-COVID-19 syndrome include fatigue, fever, and respiratory symptoms, such as difficulty breathing, shortness of breath, and coughing. SARS-CoV-2 induces cellular damage by stimulating an immune response that increases inflammatory cytokine production and pro-coagulation. Patients with severe illness have higher rates of organ damage, readmission, and death. In addition to affecting the respiratory system, COVID-19 also impacts other organs including the kidneys, liver, and heart. These effects can lead to the development of new conditions, such as heart disease, neurological symptoms, joint or muscle pain, digestive issues, blood clots, and vascular problems, as well as other symptoms such as inflammation and issues with the immune system [2]. Natural compounds have presented several targets against the SARS-CoV-2 virus, such as spike glycoprotein, papain-like protease, and nucleocapsid protein. Bormann et al. reported that turmeric extract and curcumin effectively neutralized SARS-CoV-2 at subtoxic concentrations in Vero E6 and human Calu-3 cells [3]. Curcumin, a major bioactive compound in turmeric root extract, has shown benefits for COVID-19 in terms of being antiviral, anti-inflammatory, anticoagulant, and cytoprotective [4]. Therefore, curcumin could serve as an adjuvant drug in post-COVID-19 treatment. Vahedian-Azimi et al. revealed that six studies showed that curcumin supplementation significantly decreased the severity of symptoms, duration of hospitalization, and mortality rate. The mechanism of curcumin was thought to be a significant decrease in proinflammatory cytokines such as IL-1β and IL-6 while increasing anti-inflammatory cytokines, such as IL-10, IL-35, and TGF-α [5,6,7,8,9,10].

Although curcumin has shown various inhibitory mechanisms of SARS-CoV-2 viral pathways and served as a candidate for COVID-19 adjunct treatment, the pharmacologic potential of curcumin is limited owing to poor water solubility. In addition, curcumin is rapidly metabolized after oral administration by the gastrointestinal tract and liver metabolism, reducing the bioavailability of curcumin [11]. To overcome these drawbacks and facilitate curcumin application among patients post-COVID-19, nanosized drug delivery systems have been used to protect curcumin from chemical and metabolic degradation, enhance solubility, and transport it into the cells. Sharma et al. encapsulated curcumin in polysaccharide nanoparticles and demonstrated the inhibition of cytokine, chemokine, and growth factor release associated with damage of SARS-CoV-2 spike protein stimulated liver and lung epithelial cells [12]. Tahmasebi et al. reported that nanocurcumin upregulated Treg cells and expression levels of FoxP3, IL-10, IL-35, and TGF-β, as well as the serum secretion levels of cytokines [6]. Valizadeh et al. demonstrated that nanocurcumin significantly reduced IL-6 expression and secretion levels in both serum and supernatant, as well as IL-1β gene expression and secretion levels in serum and supernatant among patients with COVID-19 [10].

In this study, nanoparticles of curcumin (nanocurcumin) were synthesized by encapsulating curcumin in phospholipids, and the particle size distribution was controlled using poloxamer 407 and polyethylene glycol 400. The resulting nanocurcumin was then subjected to investigations to evaluate its physical and chemical stability. Regarding the in vitro experiment, numerous studies have adopted various cell culture models of airway epithelial cells including human nasal epithelial cells (HNEC), human bronchial epithelial cells (HBEC), type II pneumocyte cell line (A549 cells), or lung cancer cell line (Calu-3 cells), which express the angiotensin-converting enzyme 2 (ACE2) receptor [13,14,15,16,17]. These cell cultures have been used to investigate the mechanism of spike S1 glycoprotein (CoV2-SP)-induced inflammation and conduct related experiments with SARS-CoV-2 induction [18,19]. Therefore, the study aimed to assess the anti-inflammatory properties of nanocurcumin by examining its ability to inhibit the expression of inflammatory genes (*IL-6*, *IL-1β*, *IL-18*, and *NLRP3*) and the release of cytokines (IL-6, IL-1β, and IL-18) induced by CoV2-SP. Additionally, the inhibition of NLRP3 inflammasome machinery proteins responsible for the CoV2-SP-induced inflammasome inflammatory pathway was evaluated using A549 lung epithelial cell line.

**Figure 1:**
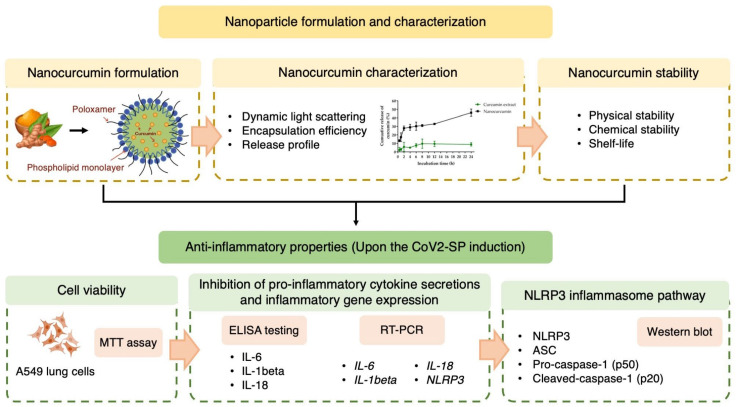
Workflow of the application of nanocurcumin formulation and characterization in inhibiting the NLRP3 inflammasome pathway for the CoV2-SP-induced inflammasome inflammatory pathway was evaluated using A549 lung cells. IL-6: interleukin-6; IL-1β: interleukin-1beta; IL-18: interleukin-18; NLRP3: nucleotide-binding oligomerization domain-like receptor containing pyrindomain 3; ASC: apoptosis-associated speck-like protein containing a caspase recruitment domain.

This study constitutes the first to demonstrate the application of nanocurcumin in inhibiting the NLRP3 inflammasome pathway in lung cells as shown in Figure 1. In addition, the method of encapsulating nanocurcumin using the solvent displacement technique with a mixture of polyethylene glycol and poloxamer as stabilizers has not been reported before, to the best of our knowledge. The formulation of nanocurcumin in this study exhibited improved physicochemical characteristics compared with curcumin extract. The release profile of nanocurcumin was also enhanced compared with curcumin extract. Moreover, nanocurcumin demonstrated an extended shelf-life of 9.7 months when stored at 4 to 5 °C, surpassing the shelf-life of curcumin in β-cyclodextrin inclusion complex (5.1 months) and curcumin in liposome (3.5 months) [20,21].

## 2. Results

### 2.1. Particle Size, Size Distribution, and Surface Charge of Nanocurcumin

To prepare the colloidal stable and redispersible nanocurcumin, we modified the solvent displacement method to successfully prepare nanocurcumin with an average hydrodynamic diameter of 136.4 ± 3.44 nm. The particle size distribution was controlled using PEO-PPO-PEO triblock-co-polymer poloxamer 407 as a nonionic surfactant and polyethylene glycol 400. The polydispersity index of freshly prepared nanocurcumin was 0.189 ± 0.015. The blank nanoparticle has a particle size of 101.33 ± 2.26 nm with a polydispersity index of 0.307 ± 0.03. The zeta potential determined the surface charge of the nanoparticles. A more negative or positive zeta potential value resulted in more stable nanoparticles. The zeta potential of blank nanoparticles was −10.09 ± 1.69 mV, while nanocurcumin had a zeta potential value of −13.47 ± 1.75 mV. 

**Figure 2:**
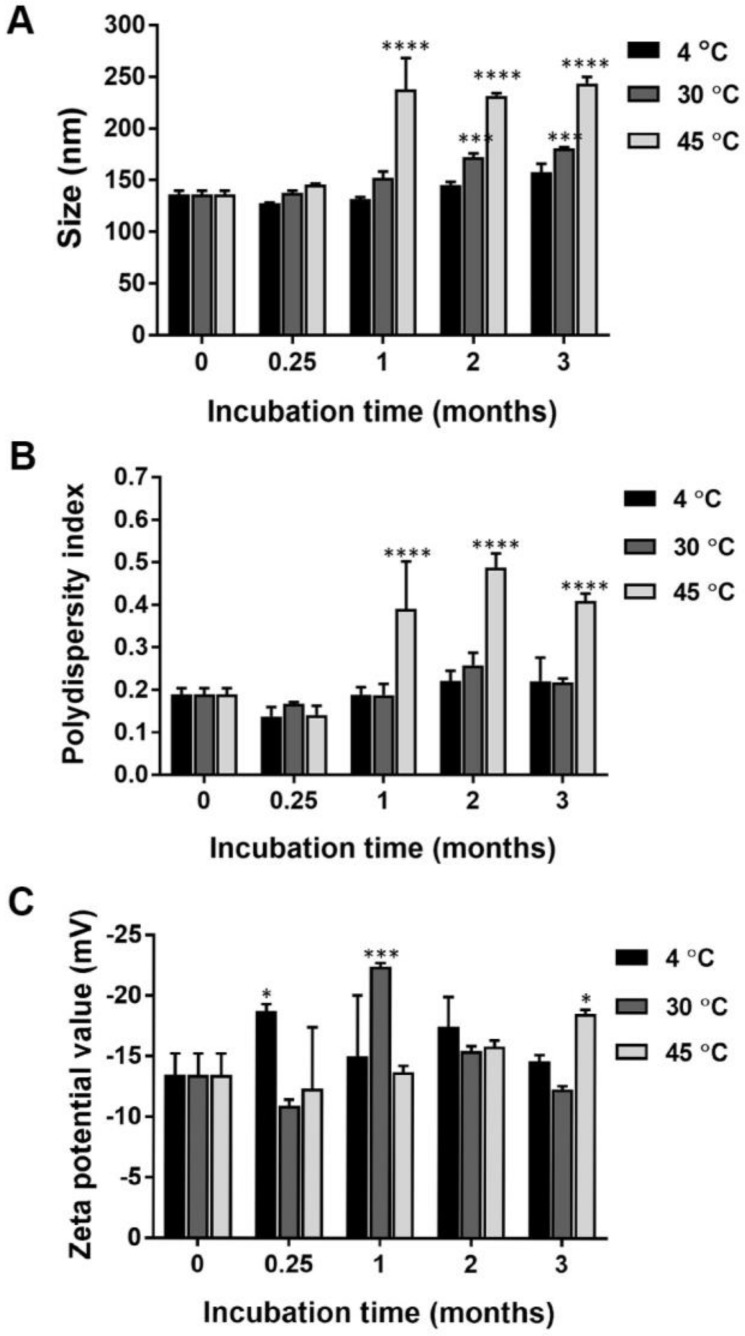
Physical stability of nanocurcumin, particle size (**A**), polydispersity index (**B**), and zeta potential of nanocurcumin (**C**) in different storage times, i.e., 0.25, 1, 2, and 3 months at 4, 30, and 45 °C. Particle size and polydispersity index were measured using the dynamic light scattering technique. Zeta potential values were analyzed by electrophoretic mobility of nanocurcumin. Data are presented as mean ± S.D. values of three independent experiments. * *p* < 0.05, *** *p* < 0.001, and **** *p* < 0.0001 compared with the freshly prepared nanocurcumin.

The size of nanocurcumin stored at 4 °C for three months was not significantly altered. The nanocurcumin had a significantly larger diameter when stored at 30 °C and 45 °C for up to two and one months, respectively (Figure 2A). The polydispersity index values of nanocurcumin stored at 4 °C and 30 °C for three months were not significantly changed, while the polydispersity index of nanocurcumin was significantly increased when the nanoparticles were kept at 45 °C for one month (Figure 2B). The zeta potential values of nanocurcumin ranged from −13.5 to −18.7 mV, −10.9 to −22.4 mV, and −12.3 to −18.5 mV when stored at 4, 30, and 45 °C, respectively (Figure 2C). The nanocurcumin was formed according to the rapid diffusion of the organic solvent due to the Marangoni effect. PEG 400 and poloxamer were used as stabilizers to effectively produce acceptable size, size distribution, and surface charge of the nanocurcumin. The stabilizing mechanisms of poloxamer include steric hindrance and repulsive hydration force on the hydrophilic surface of nanocurcumin [22]. The stability results suggested that nanocurcumin possessed optimal physical characteristics to obtain a stable colloidal system for up to three months when stored at 4 °C.

### 2.2. Encapsulation Efficiency of Nanocurcumin

**Figure 3:**
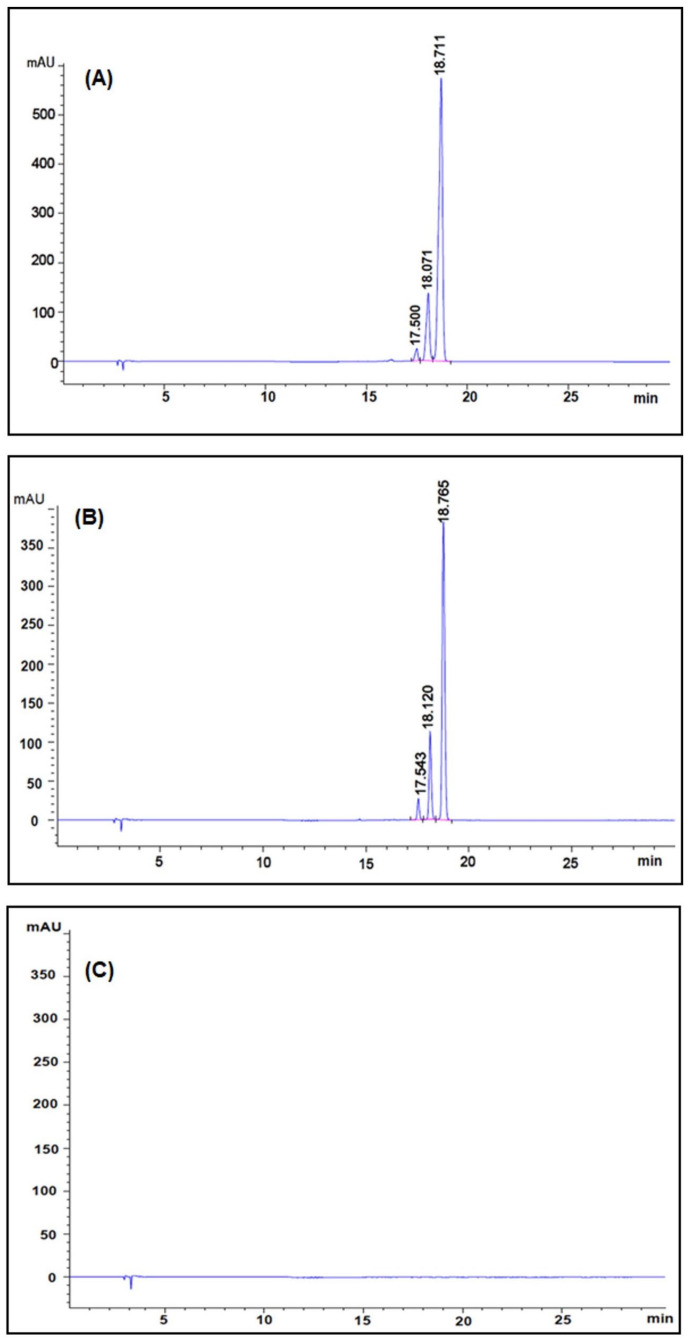
HPLC chromatograms of standard curcumins (**A**), nanocurcumin (**B**), and blank nanoparticles (**C**). The reversed phase HPLC was performed on Agilent (Series 1260, Santa Clara, CA, USA) and photodiode array (PDA) detector; C18 column (250 mm × 4.6 mm (i.d.)), 5 μm particle size; mobile phase: a linear gradient solvent system comprising acetonitrile, 0.1% phosphoric acid in water and methanol; elution time 12 min; flow rate of 1.0 mL/min and detection wavelength 425 nm.

The encapsulation efficiency of curcumin extract in nanocurcumin was determined by analyzing the curcumin content in the nanocurcumin using HPLC analysis. Figure 3A displays the chromatogram of standard curcumins, with retention times of 18.71 min for curcumin, 18.07 min for demethoxycurcumin, and 17.50 min for bisdemethoxycurcumin. Figure 3B presented the HPLC chromatogram of nanocurcumin, where the curcumin peaks were separated without any significant interference from the sample matrix. The peaks of nanocurcumin demonstrated the presence of curcumin, demethoxycurcumin, and bisdemethoxycurcumin at retention times of 18.76 min, 18.12 min, and 17.54 min, respectively. The chromatogram of blank nanoparticles confirmed the absence of curcumin content (Figure 3C), indicating successful encapsulation of curcumin in the nanocurcumin formulation. In addition, the phosphatidylcholine and surfactant used for nanocurcumin preparation did not interfere with determining curcumin. The encapsulation efficiency of curcumin in nanocurcumin was achieved by calculating the amount of curcumin present using a calibration curve (y = 118,765x + 544.3) with an r^2^ value of 0.9997, over a standard curcumin concentration range of 0.01 to 0.10 mg/mL. The results showed a high encapsulation efficiency of 90.74 ± 5.35%.

### 2.3. In Vitro Release of Curcumin from the Nanocurcumin

**Figure 4:**
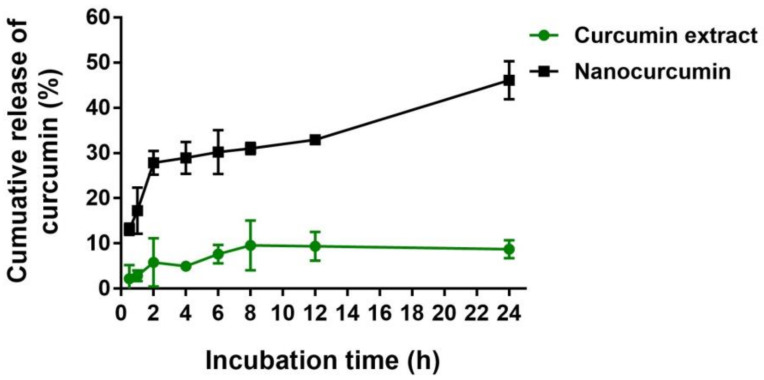
In vitro release profile of curcumin from nanocurcumin and curcumin extract in phosphate buffered solution pH 6.5 at 37 °C. The samples of nanocurcumin were collected at 15 min, 30 min, 1 h, 2 h, 4 h, 6 h, 8 h, 12 h, and 24 h. The curcumin content was analyzed using the UV–visible spectrophotometry method.

The nanocurcumin exhibited a rapid release of curcumin within the first 6 h, followed by a slow release over the remaining time period in PBS pH 6.0. The cumulative dissolution profiles of nanocurcumin are shown in Figure 4. At 6 h, 30.20 ± 4.87% of curcumin was released from nanocurcumin, while only 7.64 ± 2.03% of curcumin was released from non-nanoparticle curcumin or curcumin extract. These results suggest that nanocurcumin exhibited a higher release amount than the extract. After 24 h, the total amount of curcumin released from nanocurcumin and non-nanoparticle curcumin was 46.13 ± 4.24% and 8.73 ± 1.97%, respectively.

### 2.4. Chemical Stability of Nanocurcumin

**Figure 5:**
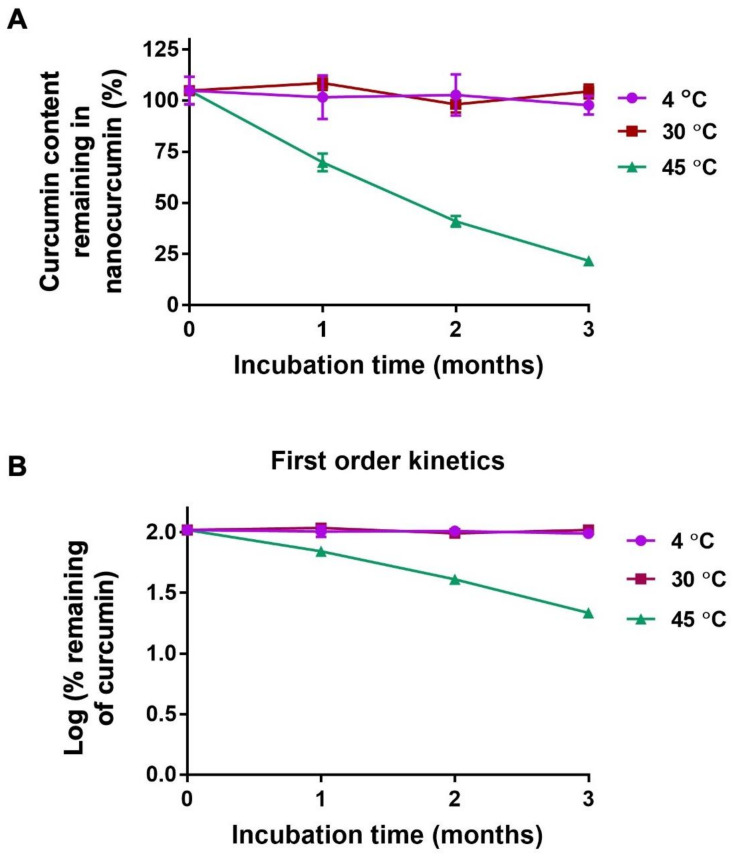
Long-term chemical stability prediction of nanocurcumin based on accelerated stability data in 3 months at intendent storage temperature. Remaining (%) of curcumin in nanocurcumin (**A**) after storage at 4, 30, and 45 °C for 3 months. Log plot of curcumin remaining in nanocurcumin (**B**) after storage at 4, 30, and 45 °C as a function of time. The curcumin concentration was analyzed using the HPLC method. Based on r^2^ determination, chemical degradation of nanocurcumin followed the first-order kinetics. The kinetic model was to predict shelf-life.

The major problem in using curcumin as a therapeutic agent is the rapid chemical degradation through hydrolysis and oxidation of curcumin in an aqueous solution at neutral pH. In this study, the stability of curcumin inside the nanocurcumin was investigated using UV–vis spectrophotometry at different time intervals at storage temperatures. The results showed that after three months of chemical stability studies, the amounts of curcumin in the nanoparticles stored at 4, 30, and 45 °C was 97.73 ± 4.58%, 108.35 ± 7.15%, and 21.69 ± 0.82%, respectively (Figure 5A). The chemical stability of curcumin might be increased by protecting it from the hydrolysis reaction at hydroxyl groups of curcumin. Encapsulating curcumin in the hydrophobic environment was considered a mechanism to protect curcumin from an aqueous environment [23].

The linear regression analysis suggested that curcumin degradation in nanocurcumin was a first-order kinetic as the plot of the log % remaining against incubation time yielded a straight line with an r^2^ of 0.1138, 0.7786, and 0.9906 for degradation at 4, 30, and 45 °C, respectively (Figure 5B). The rate constants increased with increasing storage temperatures. The shelf-lives (90% remaining) of nanocurcumin were 9.7 months, 5.12 months, and 6 days, respectively, at a storage temperature of 4, 30, and 45 °C. The nanocurcumin was recommended to be stored at 4 °C for the longest shelf-life.

### 2.5. Effect of Nanocurcumin on Cell Viability of A549 Lung Epithelial Cells

**Figure 6:**
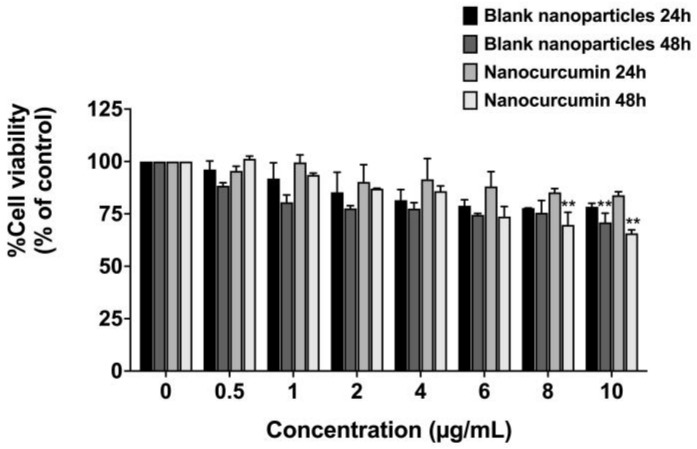
Effects of nanocurcumin and blank nanoparticles on A549 lung cells’ viability. Cells were treated with blank nanoparticles and nanocurcumin for 24 and 48 h. Cell survival was determined using an MTT assay. Data are presented as mean ± S.D. values of three independent experiments. ** *p* < 0.01 compared with the control.

Before determining the anti-inflammatory properties of nanocurcumin, the cell viability of the nanocurcumin against the A549 lung epithelial cell lines was first investigated. Using the MTT assay, as shown in Figure 6, no effect on cell viability was observed of the nanocurcumin at the concentrations of 0–10 μg/mL (inhibitory concentration at 50% cell survival or IC_50_ > 10 μg/mL) when the A549 cells were pretreated with nanocurcumin and blank nanoparticles for 24 h. Cell viability of less than 75% was observed when the A549 cells were treated with either nanocurcumin or its blank nanoparticles at 8 μg/mL and 10 μg/mL, respectively, for 48 h (Figure 6). Therefore, to investigate the anti-inflammatory properties of nanocurcumin in A549 cells, the nontoxic concentration of nanocurcumin (0–7.5 μg/mL) was chosen for further experiments.

### 2.6. Effects of Nanocurcumin on the Inhibition of Pro-Inflammatory Cytokine Secretions and Inflammatory Gene Expressions in CoV2-SP-Induced A549 Cells 

**Figure 7:**
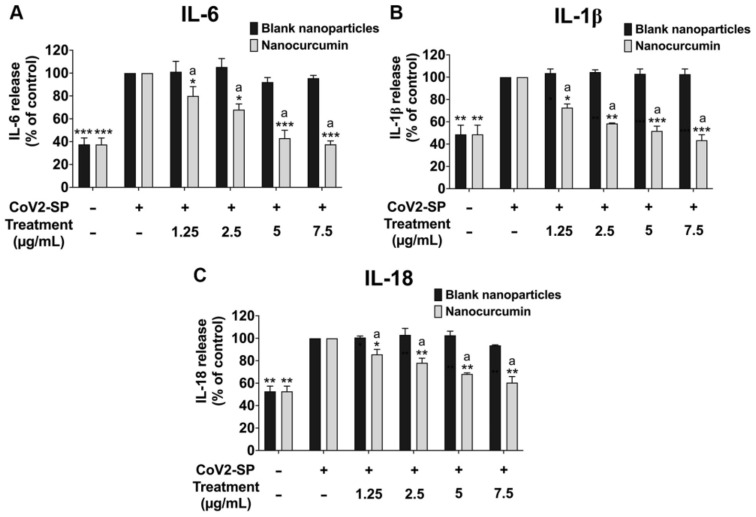
Inhibitory effects of nanocurcumin and blank nanoparticles on the pro-inflammatory cytokine secretion in CoV2-SP-induced A549 cells. A549 cells were pretreated with nanocurcumin and blank nanoparticles at the concentration of 0–7.5 μg/mL for 24 h. Then, the cells were exposed to CoV2-SP (100 ng/mL) for 3 h. The IL-6 (**A**), IL-1β (**B**), and IL-18 secretions (**C**) in the culture supernatant were examined by ELISA. The CoV2-SP-induced A549 cells are presented as 100%. Data are presented as mean ± S.D. values of three independent experiments; ** *p* < 0.01 and *** *p* < 0.001 compared with the CoV2-SP-induced control group. ^a^* *p* < 0.05, ^a^** *p* < 0.01, and ^a^*** *p* < 0.001 compared with the blank nanoparticles at the same concentration.

After obtaining the nontoxic concentrations of nanocurcumin against the A549 lung cells, we further investigated the anti-inflammation properties through the inhibition of cytokine secretions (by ELISA testing) and inflammatory gene expressions (by RT-PCR) upon the CoV2-SP induction. CoV2-SP exposure in A549 resulted in a significant increase in cytokine secretions of approximately 60% for IL-6 and a 50% increase for IL-1β and IL-18 when compared with the non-CoV2-SP-treated control group (Figure 6, *p* < 0.05). When pretreating the cells with nanocurcumin at the concentration of 1.25 to 7.5 μg/mL, the IL-6 (Figure 7A), IL-1β (Figure 7B), and IL-18 (Figure 7C) cytokine levels were significantly decreased in a dose-dependent manner when compared with the CoV2-SP-treated control group and each blank nanoparticles for the respective concentration of nanocurcumin (*p* < 0.05).

**Figure 8:**
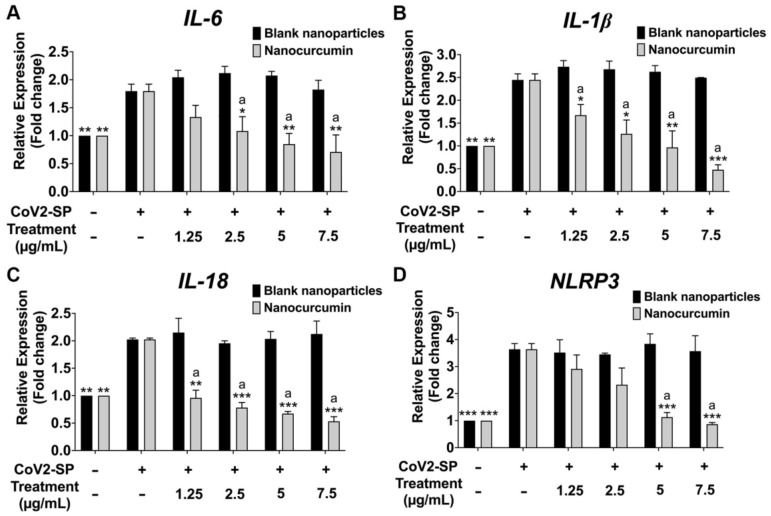
Inhibitory effects of nanocurcumin and blank nanoparticles on the IL-6 (**A**), IL-1β (**B**), IL-18 (**C**), and NLRP3 gene expression (**D**) in CoV2-SP-induced A549 cells. A549 cells were pretreated with nanocurcumin and blank nanoparticles at the concentration of 0–7.5 μg/mL for 24 h. Then, the cells were exposed to CoV2-SP (100 ng/mL) for 3 h. The mRNA expressions were determined using RT-qPCR. Data are presented as mean ± S.D. values of three independent experiments; ** *p* < 0.01 and *** *p* < 0.001 compared with the CoV2-SP-induced control group. ^a^* *p* < 0.05, ^a^** *p* < 0.01, and ^a^*** *p* < 0.001 compared with the blank nanoparticles at the same concentration.

Regarding the inhibition of inflammatory gene expression determined by RT-PCR, CoV2-SP exposure in A549 resulted in a significant increase in mRNA levels at approximately 1.8-fold for IL-6, 2-fold for IL-18, 2.5-fold for IL-1β, and 3.5-fold for NLRP3 when compared with that of the non-CoV2-SP-treated control group (Figure 8, *p* < 0.05). When pretreated, the A549 cells with nanocurcumin at the concentration of 1.25 to 7.5 μg/mL had mRNA levels of those inflammatory genes that were significantly decreased in a dose-dependent manner when compared with the CoV2-SP-treated control group and each blank nanoparticles for the respective concentration of nanocurcumin (*p* < 0.05). Overall, nanocurcumin possessed anti-inflammatory properties by inhibiting inflammatory gene expressions and inflammatory cytokine secretions. Furthermore, as these inflammatory genes and cytokines were the products of inflammasome pathway activation, we further investigated the inhibitory effects of nanocurcumin on the NLRP3 inflammasome machinery proteins in the next experiment.

### 2.7. Inhibitory Effects of Nanocurcumin on NLRP3 Inflammasome Pathway in CoV2-SP-Induced A549 Cells

**Figure 9:**
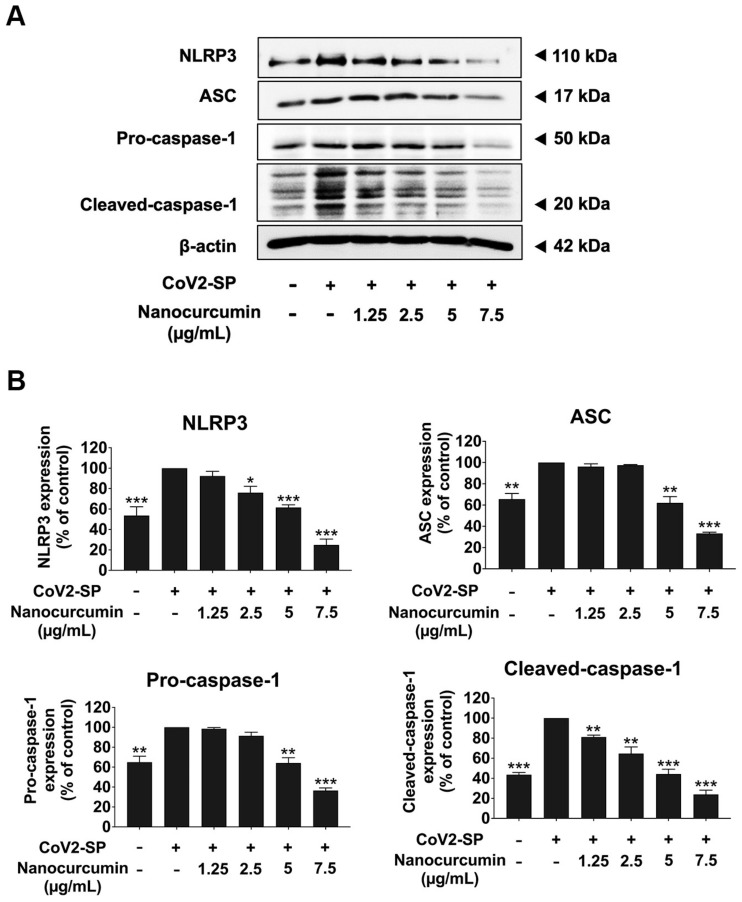
Nanocurcumin inhibited the NLRP3 inflammasome pathway in CoV2-SP-induced A549 lung cells. A549 lung cells were pretreated with the nanocurcumin at the concentration of 0–7.5 μg/mL for 24 h, then exposed with CoV2-SP (100 ng/mL) for 3 h. The inhibitory effects of nanocurcumin on the expression of NLRP3, ASC, and pro-caspase-1 and cleaved-caspase-1 proteins in A549 cells are displayed in Western blot (**A**) and band density measurements (**B**). The CoV2-SP-induced A549 is presented as 100% of the control. Data are presented as mean ± S.D. values of three independent experiments; * *p* < 0.05, ** *p* < 0.01, and *** *p* < 0.001 compared with the CoV2-SP-induced A549 cells.

As NLRP3 inflammasome is the potential inflammatory pathway for inflammation-related COVID-19, we determined whether nanocurcumin could inhibit the NLRP3 inflammasome pathway using the Western blot technique. Upon the CoV2-SP induction in A549 cells, the inflammasome machinery protein expressions, including NLRP3, ASC, and pro-caspase-1 proteins, were enhanced when compared with the non-CoV2-SP-treated control (Figure 9). Moreover, the active form of caspase-1 protein (cleaved-caspase-1), representing the activation of the inflammasome pathway, was also increased upon CoV2-SP induction when compared with the non-CoV2-SP-treated control. In pretreated A549 cells with nanocurcumin at the concentration of 1.25–7.5 ug/mL, nanocurcumin could significantly attenuate the CoV2-SP-induced inflammation via downregulation of inflammasome machinery proteins expressions (NLRP3, ASC, and Caspase-1) as well as the cleaved-caspase-1 expression in A549 cells in a dose-dependent manner (*p* < 0.05, band density measurements). Overall, it can be assumed that the inhibitory effects of nanocurcumin on CoV2-SP-induced inflammation were potentially due to the inhibition of NLRP3 inflammasome pathway activation through the suppression of those inflammasome machinery protein expression (NLRP3, ASC, and caspase-1) and cleaved form of caspase-1 in A549 cells.

## 3. Discussion

The SARS-CoV-2 virus is responsible for causing COVID-19, leading to a range of inflammatory disorders, with symptoms ranging from subchronic inflammation to severe pneumonia and acute respiratory distress syndrome (ARDS) [24]. SARS-CoV-2 is a zoonotic infection believed to have originated from bats and transmitted to humans through an intermediate animal host, possibly a wild animal sold in Chinese markets, such as camels, civets, dogs, donkeys, horses, swine, rats, deer, and pangolins [25] related studies have demonstrated that SARS-CoV-2 can elicit specific immune responses that exhibit cross-reactivity with other coronaviruses, including those responsible for common colds, such as HCoV-229E, HCoV-NL63, HCoV-OC43, and HCoV-HKU1 [26]. Therefore, continued studying and understanding the development of the SARS-CoV-2 virus in intermediate animal hosts and the potential cross-reactivity between SARS-CoV-2 and other pathogens is essential. These efforts will aid in developing effective strategies to prevent and control emerging respiratory pathogens. One of the key factors contributing to this inflammation is the virus’s spike glycoprotein (CoV2-SP), which has shown to share antigenic epitopes with human molecular chaperones [27]. When CoV2-SP activates Toll-like receptors (TLRs) and other pattern recognition receptors (PRRs), it triggers downstream inflammatory pathways and the uncontrolled release of inflammatory cytokines, leading to COVID-related inflammation [28]. Numerous studies have confirmed the role of CoV2-SP in inducing inflammation during COVID-19, with related research demonstrating that it can trigger an inflammatory response in monocyte, macrophage, and lung epithelial cell lines [29,30]. In our related studies, CoV2-SP was found to induce the release of NLRP3 cytokine, including IL-1β, IL-18, and IL-6, in both gene and protein levels in A549 lung and THP-1 macrophages cells [30,31,32]. Herein, this study aimed to investigate therapeutic approaches that target CoV2-SP-induced inflammatory responses through the NLRP3 inflammasome machinery and its cytokine products. 

Curcumin is a bioactive compound found in turmeric root extract with high potency and low toxicity. It has been shown to have significant anti-inflammatory properties, making it a promising treatment option for COVID-related inflammation. Additionally, curcumin has antiviral, anti-inflammatory, anticoagulant, and cytoprotectant properties, making it a valuable adjunct drug in post COVID-19 or long-COVID treatment [4,33]. However, curcumin’s low water solubility and bioavailability have limited its efficacy. To overcome these drawbacks and simplify the use of curcumin among patients post-COVID-19, nanoscale drug delivery methods have been developed to protect it from chemical and metabolic degradation, enhance its solubility, and facilitate its transport into cells. The use of nanocurcumin formulations has been suggested for post-COVID inflammatory syndromes due to its holistic therapeutic effects, including myocardial protection, prevention of edema formulation, anti-inflammatory properties, and antioxidant effects [34]. However, further investigation is warranted to explore its potential in this context.

In our study, nanocurcumin was formulated using phospholipids derived from soy lecithin and cholesterol. The nanocurcumin had an average hydrodynamic diameter of 136.4 ± 3.44 nm. The particle size distribution was controlled using PEO-PPO-PEO triblock-co-polymer poloxamer 407 as a nonionic surfactant and polyethylene glycol 400. The negative charge on the surface of nanoparticles (>±10 mV) increases particle repulsion, improving the colloidal stability of the nanoparticles. The ionized hydroxyl groups of PEG 400 and poloxamer 407 contribute to the negative surface charge of the nanocurcumin. Our results suggest that PEG and poloxamer-coated nanocurcumin is stable and suitable for use in biomedical applications [35]. Related studies have also reported the spherical shape of curcumin-loaded phospholipid-based nanoparticles. Hasan et al. demonstrated that the size of curcumin-nanoliposome composed of soy lecithin was 132 ± 1.3 nm, with a PDI value of 0.20 ± 0.01 [36]. Gao et al. prepared liposome containing curcumin using soy lecithin and cholesterol as phospholipids, and the liposome exhibited a spherical shape with an average size of 157.97 ± 1.41 nm [37]. Similarly, Bonaccorso et al. investigated curcumin-loaded solid lipid nanoparticles composed of a phospholipid, 2-Dimyristoyl-sn-glycero-3-phosphocholine, which had a size of 127.10 ± 11.30 nm. These nanoparticles also displayed a spherical shape, consistent with other studies [38]. Therefore, considering the use of the same materials for nanoparticle preparation and the compatibility of the particle size with related studies, it can be inferred that the nanocurcumin in our study also had a spherical shape. 

Formulating curcumin into nanocurcumin has been shown to increase its bioavailability. The increased bioavailability is primarily due to an increase in drug solubility, resulting from a reduction in particle size and an increase surface area per unit mass [39]. Smaller nanoparticles have stronger surface curvature than the larger particles, resulting in higher dissolution pressure and a higher saturation solubility [40]. An increased saturation solubility leads to an increase in the drug’s concentration gradient between the particle surface and the medium. This increased surface area and concentration gradient results in a more pronounced increase in dissolution rate compared to larger or insoluble particles of curcumin extract [41].

The HPLC analysis showed that the encapsulation efficiency of curcumin extract was 90.74 ± 5.35% in nanocurcumin. The in vitro release content of curcumin from nanocurcumin was higher than that of curcumin extract, indicating that PEG and poloxamer-coated nanocurcumin can maintain its dispersion stability and has potential applications in the biomedical field [23]. In contrast, Sharma et al. investigated the inhibitory effect of curcumin polysaccharide nanoparticles on CoV2-SP-induced cytokine storm in liver and lung epithelial cells [12]. They prepared the nanoparticles by emulsion solvent evaporation using polyvinyl alcohol as a surfactant, but the entrapment efficiency of the nanoparticles was only 25%, which is lower than the results reported here. However, the zeta potential values of the nanocurcumin were −18 mV, which matches our findings.

With regard to our in vitro investigation of the CoV2-SP-induced inflammatory mechanism, as well as the therapeutic potential of the nanocurcumin, we selected A549 cells as our cell culture model because the ACE2-expressed airway epithelial cells have been used in the experiments of related studies focusing on SARS-CoV-2 infection and inflammation [30,31,42,43]. In line with these studies, A549 cells were employed to represent the lower respiratory system or the alveolar epithelial cells that were induced by the spike glycoprotein. Consequently, the A549 cells could activate inflammatory signaling, resulting in the release of pro-inflammatory cytokines. The inflammasome complex is composed of NLRP3, ASC, and zymogen pro-caspase-1 machinery proteins, which predominantly respond to intracellular damage induced by pathogens. Once the protein complex has formed, pro-caspase-1 is cleaved, and inflammasomes activate caspase-1 (cleaved caspase-1), cleaving pro-IL-1β and pro-IL-18, converting them into their biologically active forms, leading to the secretion of potent pro-inflammatory cytokines [44,45]. NLRP3 is known to coordinate the uncontrolled inflammatory pathway referred to as the NLRP3 inflammasome machinery and is one of the most detrimental signaling molecules in chronic lung inflammatory conditions [46]. The spike glycoprotein from SARS-CoV-2 has been demonstrated to activate TLRs, leading to the activation of various inflammatory signaling pathways (MAPK and JAK-STAT) and transcription factors such as NF-kB and AP-1. This results in the production of inflammatory genes such *IL-6*, *IL-1β*, and *IL-18* [47,48,49,50].

In this study, we found that nanocurcumin inhibits NLRP3 inflammasome activation induced by CoV2-SP by downregulating the inflammasome component protein expressions (NLRP3, ASC, and pro-caspase-1). To confirm the anti-inflammatory properties of nanocurcumin, we measured the mRNA levels of inflammatory gene expressions (*NLRP3*, *IL-6*, *IL-1β*, and *IL-18*), the protein expression of inflammasome components in cell lysate, and cytokine release of IL-1β and IL-18 in the cell culture supernatant. Our results showed that CoV2-SP exposure in A549 lung cells increased the expressions of inflammatory genes (*NLRP3*, *IL-6*, *IL-1β*, and *IL-18*) and cytokine releases (IL-6, IL-1β, and IL-18). However, nanocurcumin suppressed the inflammatory response induced by CoV2-SP at the gene and cytokine levels of the NLRP3 inflammasome pathway. Therefore, nanocurcumin could be a potential therapeutic agent for the treatment of lung inflammatory conditions induced by CoV2-SP.

## 4. Materials and Methods

### 4.1. Materials

The standard curcumin compound was purchased from Merck Millipore (Billerica, MA, USA). Curcumin extract (≥70% purity, HPLC) was obtained from Welltech Biotechnology Co., Ltd. (Bangkok, Thailand). Phosphatidylcholine, cholesterol, and poloxamer 407 were obtained from Chanjao Longevity Co., Ltd. (Bangkok, Thailand). A recombinant human coronavirus SARS-CoV-2 spike glycoprotein S1 (ab273068) was purchased from Abcam company (Cambridge, UK). Dulbecco’s Modified Eagle Medium (DMEM) was purchased from Gibco (Grand Island, NY, USA). The fetal bovine serum was purchased from Thermo Scientific Company (Waltham, MA, USA). The MTT or 3-(4,5-dimethylthiazol-2-yl)-2,5-diphenyltetrazolium bromide dye and mouse anti-beta-actin primary antibody were purchased from Sigma-Aldrich Company (St. Louis, MO, USA). Rabbit anti-NLRP3 primary monoclonal antibody, anti-ASC monoclonal antibody, anti-pro-caspase-1 (p50) monoclonal antibody, anti-cleaved-caspase-1 (p20) monoclonal antibody, and goat horseradish peroxidase-conjugated anti-mouse- or anti-rabbit-IgG were obtained from Cell Signaling Technology company (Danvers, MA, USA). The TRI reagent^®^ was purchased from Merck Millipore Company (Billerica, MA, USA). The ReverTra Ace^®^ qPCR Master Mix was purchased from Toyobo Co., Ltd. (Osaka, Japan). The SensiFAST SYBR Lo-ROX Kit was purchased from Meridian Bioscience^®^ Company (Cincinnati, OH, USA).

### 4.2. Preparation and Characterization of Nanocurcumin

Curcumin extract (10 mg) was dissolved in acetone (10 mL). The curcumin extract solution was mixed with phosphatidylcholine (150 mg) in 95% ethanol 10 mL and cholesterol (20 mg) in acetone 2 mL. The mixture solution was infused dropwise in 80 mL deionized water containing 10 mL polyethylene glycol 400 and 10 mL of 1% poloxamer 407 with a flow rate of 1 mL/min under a stirring rate of 600 rpm. The mixture was stirred overnight to remove the organic solvent, followed by dialysis against deionized water for 2 h. The hydrodynamic diameter, polydispersity index, and zeta potential values of nanocurcumin were measured by dynamic light scattering (DLS) performed at a 173° scattering angle and at 25 °C (Zetasizer Nanoseries, Malvern instruments, Malvern, UK).

### 4.3. Physical and Chemical Stability of Nanocurcumin

The long-term stability of nanocurcumin was examined by placing samples of nanocurcumin at 4, 30, and 45 °C in a tight container for three months. Nanocurcumin was withdrawn at 0-, 1-, 2-, and 3-month intervals and evaluated for encapsulated curcumin content by a UV-vis spectrophotometry at a maximum wavelength of 435 nm, and for particle size, the polydispersity index and zeta potential were evaluated by the dynamic light scattering technique (Zetasizer ZS, Malvern, UK) to monitor the effect of the storage condition on the physical stability of nanocurcumin.

### 4.4. In Vitro Release of Curcumin

The drug release study was carried out using a Transwell^®^ insert at 37 °C. The drug release method was modified from a related study [23]. Twenty-four-well Transwell^®^ plates with polycarbonate membrane inserts and 0.4 μm membrane pore size were used. The curcumin extract (200 μL, 1 mg/mL in PBS pH 6.5) and nanocurcumin (200 μL, 1 mg/mL) were placed onto the semi-permeable Transwell^®^ membrane. A volume of 1.0 mL of preheated PBS, pH 6.4, was added to the receptor compartment. Samples of 50 μL were taken from the receptor compartment at pre-specified timepoints of 15 min, 30 min, 1 h, 2 h, 4 h, 6 h, 8 h, 12 h, and 24 h and substituted with 50 μL of fresh preheated release medium to maintain the sink conditions. All samples were quantified for curcumin content by UV-vis spectrophotometry. The concentrations of curcumin released from the extract and the nanocurcumin were calculated using the curcumin standard curve. 

### 4.5. Identification and Quantification of Curcumin Content in Nanocurcumin Using High-Performance Liquid Chromatography (HPLC)

The concentration of curcumin was determined by a modified high-performance liquid chromatography method [51]. The HPLC system by Agilent (Series 1260, CA, USA) and photodiode array (PDA) detector were used to determine the bioactive compounds in nanocurcumin. Chromatographic separation was performed on a reversed-phase C18 column (250 mm × 4.6 mm (i.d.), 5 μm particle size) provided by Mightysil, Kanto Chemical Co., Tokyo, Japan. The mobile phase consisted of (A) acetonitrile, (B) 0.1% phosphoric acid in water, and (C) methanol. The mobile-phase composition was kept constant at 8.25% A, 83.5% B, and 8.25% C for 7 min followed by a linear gradient solvent system: 0–12 min, 8.25–55% A, 83.5–40% B, and 8.25–55% C; 12–30 min, 55–8.25% A, 40–83.5% B, and 55–8.25% C (Table 1). The mobile phase was changed to the initial condition at 2 min, followed by an equilibration time of 10 min. The PDA detector was set at a wavelength of 425 nm. The flow rate was set at 1.0 mL/min, and the injection volume was 20 μL for all samples and reference standards. A 0.45 μm membrane filter was used to filter samples before HPLC analysis. The amounts of curcumin were calculated by the peak areas using a calibration curve constructed from 0.01 to 0.1 mg/mL of standard curcumin. Unloaded nanoparticles were used as a reference. In this study, the encapsulation efficiency of curcumin was analyzed directly by measuring the amount of encapsulated curcumin in nanocurcumin after the ethanolic extraction of nanocurcumin. The encapsulation efficiency of the nanocurcumin was calculated from the equations below.

$$Encapsulation\;efficiency\;\left(\%\right)=\frac{Amount\;of\;curcumin\;encapsulated}{Initial\;amount\;of\;curcumin\;added}\times 100\%$$

### 4.6. Cell Viability Testing

A549 lung epithelial cell line was obtained from American Type Culture Collection (ATCC). The cell lines were cultured in DMEM supplemented with 10% FBS, 2 mM L-glutamine, 50 U/mL of penicillin and 50 μg/mL of streptomycin. The cells were maintained in a 5% CO_2_ humidified incubator at 37 °C. 

The cell viability of the nanocurcumin and blank nanoparticles against A549 cells was determined using an MTT assay. Briefly, the A549 cells (3 × 10^3^ cells/well) were treated with increasing concentrations of nanocurcumin and blank nanoparticles (0–10 μg/mL) in a culture medium for 24 and 48 h. Then, the cells were incubated with 10 μL of 0.5 mg/mL MTT in PBS for 4 h. The culture supernatant was then removed, and the culture was re-suspended with 200 μL of DMSO to dissolve the MTT formazan crystals. The absorbance was measured at 540 and 630 nm using a UV-visible spectrophotometer. The assay was performed in triplicate at each concentration. Cell viability was calculated compared to control and interpreted as the % of the control.

### 4.7. Inhibition of Cytokine Releases by ELISA Test

The secretions of IL-6, IL-1β, and IL-18 into the cultured medium were determined using an ELISA kit (Biolegend, San Diego, CA, USA) as followed according to the manufacturer’s instruction. A549 cells were seeded in a 6-well plate. After that, the cells were pretreated with nanocurcumin (0–7.5 μg/mL) or blank nanoparticles for 24 h and then exposed to CoV2-SP at the concentration of 100 ng/mL for 3 h. The cultured medium was collected for ELISA testing, and the absorbance was measured at 450 and 570 nm. The cytokine secretions in the cultured medium were calculated and compared for each standard curve.

### 4.8. Expression of IL-6, IL-1β, IL-18, and NLRP3 Genes by RT- qPCR Analysis

To determine inflammatory gene expressions (*IL-6*, *IL-1β*, *IL-18*, and *NLRP3*), A549 cells were pretreated with nanocurcumin (0–7.5 μg/mL) or blank nanoparticles for 24 h and then exposed to 100 ng/mL of CoV2-SP for 3 h. Then, the total mRNA was isolated using TRI reagent^®^. The concentration and purity of total RNA were detected using NanoDrop™ 2000/2000c Spectrophotometers (Thermo Fisher Scientific, Waltham, MA, USA). The cDNA was obtained via reverse transcription using a Mastercycler^®^ nexus gradient machine (Eppendorf, GA, Germany). Quantitative real-time PCR technique was determined using a qRT-PCR ABITM 7500 Fast & 7500 Real-Time PCR machine (Thermo Fisher Scientific, Waltham, MA, USA). Gene expressions were analyzed using QuantStudio 6 Flex real-time PCR system software (Applied Biosystems, Waltham, MA, USA). The 2^−ΔΔCT^ method with normalization to GAPDH and controls was used for the calculation of results.

All primer sequences used in this study were as follows: IL-6 forward: 5′-ATG AAC TCC TTC ACA AGC-3′, reverse: 5′-GTT TTC TGC CAG TGC CTC TTT G-3′ (Bio Basic Canada Inc., Ontario, Canada); NLRP3 forward: 5′-AAC ATG CCC AAG GAG GAA GA-3′, reverse: 5′-GGC TGT TCA CCA ATC CAT GA-3′; IL-1β forward: 5′-TGC TCA AGT GTC TGA AGC AG-3′, reverse: 5′-TGG TGG TCG GAG ATT CGT AG-3′; IL-18 forward: 5′-TCG GGA AGA GGA AAG GAA CC-3, reverse: 5′-TTC TAC TGG TTC AGC AGC CA-3′; and GAPDH forward: 5′-TCA ACA GCG ACA CCC AC-3′, reverse: 5′-TCA ACA GCG ACA CCC AC-3′ (Humanizing Genomics Macrogen, Geumcheon-gu, Seoul, Republic of Korea) [52,53,54].

### 4.9. Western Blot Analysis

To determine the effects of nanocurcumin on inflammasome machinery proteins in CoV2-SP-induced inflammation, A549 cells were pretreated with nanocurcumin (0–7.5 μg/mL) for 24 h and then exposed to 100 ng/mL of CoV2-SP for 3 h. Then, cells were collected and lysed using RIPA buffer. The protein concentration was determined using the Bradford method. The whole-cell lysate was subjected to 12% SDS-PAGE. Separated proteins were transferred to nitrocellulose membranes. Membranes were blocked with 5% nonfat dried milk protein in 0.5% TBS-Tween. After that, the membranes were washed twice with 0.5% TBS-Tween. Then, membranes were further incubated overnight with the primary antibody at 4 °C. Next, the membranes were washed five times with 0.5% TBS-Tween followed by incubating with horseradish peroxidase-conjugated anti-mouse or -rabbit-IgG depending on the primary antibody at room temperature for 2 h and were then washed five times with 0.5% the TBS-Tween. Bound antibodies were detected using the chemiluminescent detection system and then exposed to iBright™ CL-1500 imaging system (Thermo Fisher Scientific, Waltham, MA, USA). Equal values of protein loading were confirmed as each membrane was stripped and re-probed with an anti-β-actin antibody. Band density levels were analyzed using IMAGE J 1.410.

### 4.10. Statistical Analysis

All data are presented as mean ± standard deviation (S.D.) values. Statistical analysis was analyzed with Prism Version 8.0 Software using independent *t*-test and one-way ANOVA with Dunnett’s test. Statistical significance was determined at * *p* < 0.05, ** *p* < 0.01, and *** *p* < 0.001.

## 5. Conclusions

This study developed a nanoparticle preparation of curcumin from curcumin extract to enhance its physical and chemical stability and to be used for anti-inflammatory purposes. The nanocurcumin was found to inhibit CoV2-SP-induced inflammation in A549 lung cells by inhibiting the NLRP3 inflammasome pathway (NLRP3, ASC, and Caspase-1) as well as the cleaved-caspase-1 proteins. This led to the suppression of inflammatory cytokines releases such as IL-6, IL-1β, and IL-18. Our findings provide substantial evidence supporting the use of nanocurcumin in developing supportive therapeutics to prevent COVID-19-related inflammation.

## Figures and Tables

**Table 1:** HPLC gradient program.

Time (min)	A	B	C
0	8.25	83.50	8.25
7	8.25	83.50	8.25
12	55.00	40.00	5.00
27	55.00	40.00	5.00
28	8.25	83.50	8.25
30	8.25	83.50	8.25

## Data Availability

Not applicable.
